# SIP‐SRS Imaging of Cell Wall Synthesis Identifies a Synergy between Micafungin and Amphotericin B

**DOI:** 10.1002/advs.202507331

**Published:** 2025-10-21

**Authors:** Meng Zhang, Yuewei Zhan, Haonan Lin, Jiyang Chen, Mohamed N. Seleem, Michael Mansour, Ji‐Xin Cheng

**Affiliations:** ^1^ Department of Electrical & Computer Engineering Boston University Boston MA 02215 USA; ^2^ Boston University Photonics Center Boston MA 02215 USA; ^3^ Department of Biomedical Engineering Boston University Boston MA 02215 USA; ^4^ Department of Biomedical Sciences and Pathobiology, Virginia‐Maryland College of Veterinary Medicine Virginia Polytechnic Institute and State University Blacksburg VA 24061 USA; ^5^ Harvard Medical School Boston MA 02215 USA; ^6^ Department of Chemistry Boston University Boston MA 02215 USA

**Keywords:** antifungal resistance, cell wall synthesis, fungi, single‐cell imaging, stimulated Raman scattering

## Abstract

*Candida* species causes life‐threatening infections in immunocompromised individuals and presents a formidable challenge in clinical practice. This challenge is exacerbated by the growing prevalence of drug resistance, particularly against the last resort antifungals such as amphotericin B (AmB). The fungal cell wall, known for its dynamic reorganization in response to growth demands and host threats, are recognized as a key drug target. In this study, stable isotope probe‐assisted SRS microscopy (SIP‐SRS) is harnessed to directly visualize and interrogate the dynamics of fungal cell wall synthesis under various antifungal treatments. A striking observation is the thickening of the cell wall in newly synthesized daughter cells under AmB treatment. Based on this finding, a synergistic inhibition of fungal growth is demonstrated by AmB and micafungin, an antifungal agent targeting cell wall synthesis. These results not only advance the understanding of fungal physiology at the molecular level but also open promising avenues for combating drug‐resistant fungal infections.

## Graphical abstract

We employed glucose‐d7–based stable isotope probe‐assisted SRS microscopy (SIP‐SRS) C–D imaging to visualize fungal cell wall synthesis and remodeling under antifungal treatment. Amphotericin B (AmB) induced notable daughter cell wall thickening, prompting a combinational therapy with AmB and micafungin. This approach synergistically inhibited fungal growth, revealing therapeutic potential through targeted cell wall remodeling analysis.

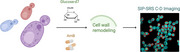

## 1 Introduction

Fungal infections, particularly invasive candidiasis, affects millions of patients annually and has become an emerging crisis worldwide.^[^ ^1^ ^]^ *Candida* species, one of the most significant opportunistic fungal pathogens, pose a global public health threat.^[^ ^2^ ^]^ Several *Candida* species, including *Candida albicans* (*C. albicans*) and *Candida auris (C. auris)*, cause opportunistic fungal infections and are responsible for 70% to 90% of all invasive fungal infections.^[^ ^3^ ^]^ Early treatment with appropriate antifungal drugs is the key to reduce the mortality of severe candidiasis. Common antifungals that are used to treat invasive fungal infections either target fungal membrane sterols (polyenes), inhibit the synthesis of ergosterol (azoles), or inhibit the synthesis of β‐glucan in the fungal cell wall (echinocandins).^[^ ^4^ ^]^ Unfortunately, the widespread use of antifungals has resulted in increased antifungal resistance among different fungal species, which is an emerging threat to public health.^[^ ^5^ ^]^ Fungal infections present a formidable challenge in clinical practice, exacerbated by the growing prevalence of drug resistance, particularly against antifungals such as amphotericin B (AmB). Despite intensive research efforts aimed at developing new antifungal agents and understanding resistance mechanisms, effective therapeutic solutions remain elusive.

AmB, despite its toxicity, has served as a last‐resort antifungal with potent fungicidal activity against *Candida* infections for decades. However, the emerging resistance of *C. auris* isolates to AmB poses a significant threat of treatment failure for many patients. Numerous efforts have been made to enhance the efficacy and reduce the toxicity of AmB through the development of novel formulations. An alternative strategy involves combining AmB with potent enhancers, which may effectively overcome resistance without increasing the toxicity of AmB.^[^ ^6^ ^]^

Recent advances have also explored alternative approaches, such as the clinical translation of micafungin, with pivotal studies shedding light on its mechanism of action.^[^ ^7^ ^]^ However, significant gaps remain in understanding the molecular mechanisms underpinning these therapies and the adaptive responses of fungi to combat treatment strategies.

Fungal cell walls are dynamic organelles that are essential for cell viability, morphogenesis, and pathogenesis.^[^ ^8^ ^]^ In most fungal species the inner skeletal cell wall layer is composed of chitin, β‐1,3‑glucan and β‐1,6‑glucan. This branched β‐1,3‑ and β‐1,6‑glucan is bound to proteins and/or other polysaccharides, whose composition may vary with the fungal species. In Candida species, the outer layer of the cell walls is heavily enriched with highly mannosylated proteins that are mostly attached via glycosylphosphatidylinositol remnants to β‐1,6‐glucan and to the β‐1,3‐glucan‐chitin core. The cell wall composition is highly regulated in response to environmental conditions and external stresses. Cell wall structures can dynamically reorganize to accommodate growth demands and defend against host threats. Since the cell wall is absent in mammals, it has long been recognized as an attractive target for antifungal drug development.^[^ ^9^ ^]^ This dynamic nature underscores the need for advanced imaging techniques to unravel the underlying processes and enhance our understanding of fungal cell wall adaptations.

Techniques such as stable isotope probing (SIP) allow for the precise tracking of isotopically labeled substrates within microbial cells, providing insights into metabolic pathways and substrate utilization.^[^ ^7^, ^10^ ^]^ By incorporating stable isotopes like ^13^C, D (^2^H), and ^15^N, SIP enables investigation of metabolic activities in a targeted and quantitative manner, offering a window into how microbiomes metabolize and respond to environmental changes and drug exposure.^[^ ^10^, ^11^ ^]^ Raman‐based SIP techniques have been hindered by their slow detection rates and low sensitivity to subtle metabolic changes, which can obscure real‐time metabolic insights and limit the scope of dynamic observations. These drawbacks have constrained real‐time observations of dynamic metabolic processes in complex biological systems.

To address the low speed issue in Raman‐based SIP technique, SIP aided stimulated Raman scattering (SIP‐SRS) imaging has emerged for meticulous metabolic investigations using isotopic tracers like D_2_O and glucose‐d7^[^ ^12^ ^]^ and has demonstrated success in elucidating metabolism in bacteria, mammalian cells and animal models^[^ ^12^, ^13^ ^]^ Despite these advances, SIP‐SRS imaging of cell wall remodeling, a critical target of antifungal activity, remains underexplored.

In this study, we harness glucose‐d7 based SIP‐SRS to directly visualize fungal cell wall synthesis and interrogate cell wall remodeling under antifungal treatment. By leveraging the high sensitivity and spatial resolution of SIP‐SRS, we uncovered previously inaccessible details of metabolic interaction with antifungals. A striking observation was the thickening of the daughter cell wall after AmB treatment. This finding led to the development of a combinational AmB and micafungin treatment for synergistic inhibition of fungal growth. These results not only advance our understanding of fungal physiology at the molecular level but also opens promising avenues for combating drug‐resistant fungal infections. By elucidating the intricate interplay between antifungal medications and fungal metabolic responses, our study contributes to ongoing efforts of developing effective treatments against resilient fungal pathogens.

## 2 Results

### 2.1 SIP‐SRS Imaging Reveals Newly Synthesized Cell Wall via Glucose‐d7 Metabolism

**Figure 1:**
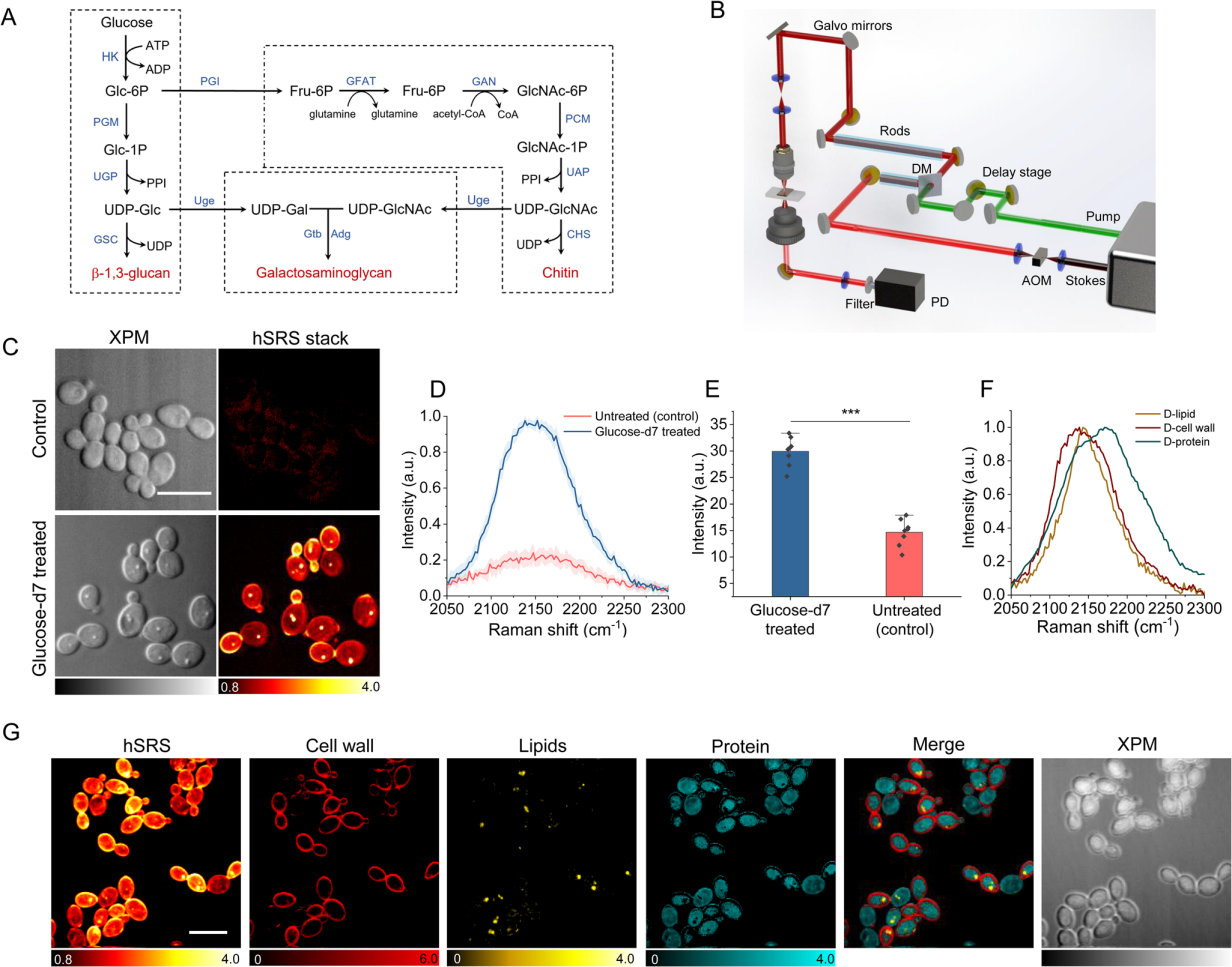
SIP‐SRS imaging of newly synthesized cell wall via glucose‐d7 metabolism. A) Scheme of cell wall polysaccharides synthesis via glycolysis in *C. albicans*.^[^ ^14^ ^]^ B) Schematic illustration of the SRS setup. AOM: acousto‐optic modulation. DM: dichroic mirror. PD: photodiode. C) SRS and corresponding XPM images of *C. albicans* after culture in normal and glucose‐d7‐containing medium for 1 h. Scale bar: 10 µm. D) Average SRS spectra of individual *C. albicans* cells after culture in normal and glucose‐d7‐containing medium. N ≥ 15 per group. E) Average SRS C–D intensity plot for *C. albicans* in C) with N ≥ 10 per group. Significance was evaluated using an unpaired *t*‐test (***, *p* < 0.001). F) Reference spectra for hSRS spectra unmixing analysis using LASSO. G) The decomposed hyperspectral SRS images into cell wall, deuterated lipids, and deuterated protein channels, and the corresponding XPM images of *C. albicans*. Scale bar: 10 µm.

Glucose is a primary source of energy for *C. albicans* to synthesize its cell wall through glycolysis (**Figure** **1**A).^[^ ^14^ ^]^ The fungal cell wall is composed mainly of β‐glucan, chitin, and glycoproteins, with the proportions of these components dynamically changing in response to environmental conditions.^[^ ^15^ ^]^ We first validate that glucose‐d7, a glucose isotopologue with deuterium labelling on all its carbons, can be used as a metabolic probe for SRS imaging. This approach enables precise visualization of fungal cell wall synthesis, providing critical insights into the dynamic remodeling of the cell wall under varying conditions. The schematic of our SRS microscope is shown in Figure 1B. In brief, the spatially and temporally overlapped pump and Stokes pulses are tuned to match the vibrational frequency of Raman‐active modes. The SRS signal appears as an intensity gain in the Stokes beam and an intensity loss in the pump beam, which is extracted through a lock‐in amplifier. In our setup, the stimulated Raman loss is measured, in which most excitation power is in the 1040‐nm Stokes beam having a high cellular damage threshold. The carbon‐deuterium (C–D) vibrational band, located in the cell‐silent region (1800–2600 cm^−1^), is spectrally differentiated from endogenous Raman bands, enabling selective detection using SRS generated by chirped femtosecond laser pulses. Specifically, strong C–D signals in *C. albicans* SC5314 can be observed after incubation in glucose‐d7‐substituted medium within 1 h via hyperspectral SRS imaging in the C–D stretching vibrational region (2050–2300 cm^−1^), confirming the incorporation of deuterium to form C–D bonds in individual cells (Figure 1C,D). Cell morphology was visualized using cross‐phase modulation (XPM) images. As a control, minimal SRS C–D signals were observed in cells cultured in normal medium (Figure 1C,D). The C–D signal in both the control group and the non‐cell regions of glucose‐d7‐treated samples was much lower than in the cellular regions of the glucose‐d7‐treated group, confirming that the observed signals are specific to newly synthesized biomolecules. To verify that the signals arise from metabolic activity, we compared conditions where cellular metabolism was active versus inhibited. Live *C. albicans* SC5314 cells cultured in glucose‐d7‐containing medium at 30 °C exhibited increasingly strong C–D signals over time (Figure S1, Supporting Information), consistent with active metabolism and incorporation of deuterium into newly synthesized biomolecules. In contrast, cells incubated at 4 °C, where metabolic activity is suppressed, showed negligible C–D signals, indicating that no significant abiotic H–D exchange occurs on preexisting C–H bonds. In a control group cultured without glucose‐d7, no C–D signals were detected. Importantly, all cell samples were thoroughly washed with PBS multiple times prior to SRS imaging to remove any residual free glucose‐d7. This step ensures that the detected C–D signals represent intracellular incorporation into macromolecules, not extracellular or unincorporated glucose. Collectively, these experiments confirm that the C–D signals are predominantly a result of deuterium incorporation into newly synthesized biomolecules through metabolic processes.

Newly synthesized daughter cells were identified based on their smaller size, budding morphology, and spatial attachment to larger mother cells.^[^ ^16^ ^]^ In budding yeast, cells divide asymmetrically by orienting the spindle perpendicularly to the site of cell division (bud neck) during the early stages of the cell cycle.^[^ ^17^ ^]^ Interestingly, the daughter cells showed stronger SRS signal contrast than the mother cells, indicating that the C–D signals originated from newly synthesized macromolecules (Figure S2, Supporting Information). We examined the budding area between the mother cells and the daughter cells which showed strong C–D intensities. We then acquired fluorescence imaging after labeling multiple layers in the cell wall of *C. albicans* (Figure S3, Supporting Information). The fluorescence image of chitin is remarkably strong between the mother cells and the daughter cells, while the mannan layer is a uniform ring shape in individual fungal cells. Further quantification analysis of single cell indicated distinctly higher C–D intensities at the glucose‐d7 treated cell than the untreated cells (Figure 1E). These results were further confirmed by spontaneous Raman spectra (Figure S4, Supporting Information), showing a broad peak (2050–2300 cm^−1^) at C–D vibration for *C. albicans* cultured in glucose‐d7‐substituted medium. The single cell SRS spectra from the cell wall region, cytoplasmic region, and the lipid droplets regions are largely different (Figure S5, Supporting Information).

To reveal the specific chemical mapping at the single cell level, we performed spectral unmixing using a pixel‐wise least absolute shrinkage and selection operator (LASSO) regression algorithm. This method applies a sparsity constraint during spectral decomposition, enabling the selective inclusion of only the most relevant reference spectra at each pixel. Specifically, pixel‐wise LASSO unmixing effectively suppresses background signals and reduces cross‐talk between channels by leveraging a sparsity constraint, which limits contributions to only the most relevant spectral components while preserving the accuracy of the reference spectra. This technique has been previously validated in our earlier work using well‐characterized model systems,^[^ ^18^ ^]^ demonstrating robust performance in high‐dimensional spectral imaging applications. The reference spectra for fungal cell wall (D‐cell wall), deuterated lipids (D‐lipids) and proteins (D‐protein) clearly showed spectral difference in the C–D stretching vibrational region (Figure 1F). The reference spectral profiles were derived from hyperspectral SRS images of glucose‐d7‐treated cells. To ensure a clean reference, the D‐protein spectrum was obtained from cell‐extracted cytoplasmic proteins. The D‐lipid and D‐cell wall spectra were obtained by averaging signals from cytoplasmic lipid droplets (mainly triglycerides) and cell wall regions in the hyperspectral SRS data, as these structures are spatially distinct and can be reliably identified. Both the D‐lipid and D‐cell wall spectra were normalized against the corresponding XPM background spectrum for LASSO unmixing. By applying spectral unmixing, the SRS C–D metabolic imaging was simultaneously decomposed into distinct channels for cell wall, deuterated lipids and deuterated protein channels (Figure 1G). Since pixel‐wise LASSO unmixing uses a sparsity constrain to prevent spectral cross‐talk and retains the accuracy of the reference spectra, there is no obvious cross‐talk between channels.^[^ ^18^ ^]^ This method leverages reference spectra obtained from metabolically labeled cells to resolve overlapping signals and achieve molecular specificity. The cell wall levels are distinguished from other biomolecules. Specifically, we utilize glucose‐d7 as a metabolic precursor, which is taken up by fungal cells and enters the glycolytic pathway. As a universal carbon source, glucose supports synthesis of diverse biomass including cell wall polysaccharides, lipids, and proteins.^[^ ^19^ ^]^ Compared with labelling cell cultures using D_2_O incorporation, it is much more difficult to investigate cell wall synthesis in individual fungal cells since water is ubiquitously used in multiple macromolecule biosynthesis pathways (Figure S6, Supporting Information). Together, SIP‐SRS imaging at C–D vibrational region provides a good means to monitor newly synthesized cell wall in a single fungal cell. Our approach is extremely sensitive to monitor cell wall dynamics at the single‐cell level.

### 2.2 Real‐Time SIP‐SRS Imaging of Cell Wall Synthesis

**Figure 2:**
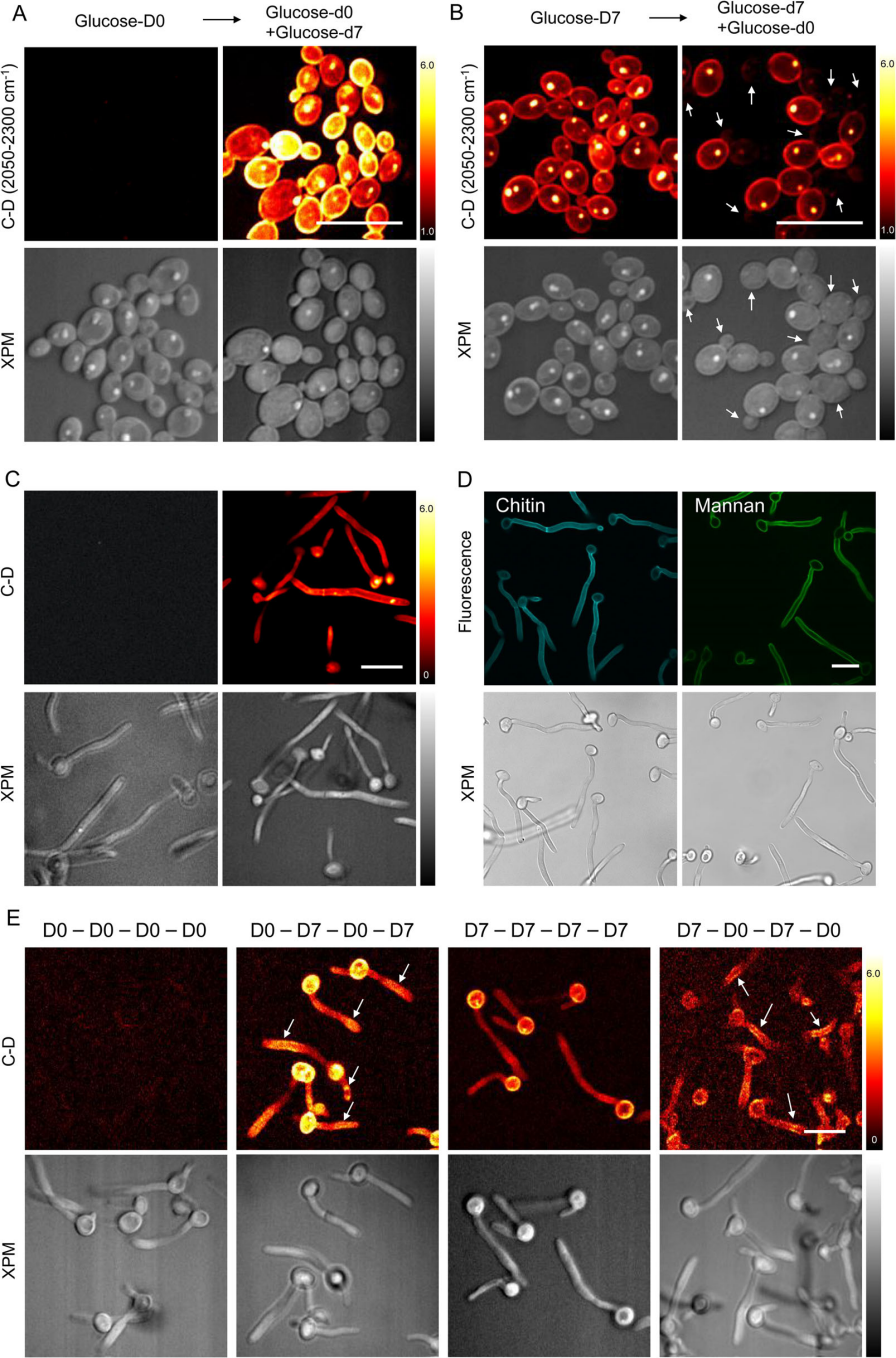
Real‐time SIP‐SRS imaging of cell wall synthesis dynamics. A) SIP‐SRS images showed non‐detectable C–D signal in the glucose‐d0‐treated *C. albicans* cells but stronger C–D signal in the glucose‐d7‐treated *C. albicans* cells. The daughter cells showed stronger cell wall signals than the mother cells after refreshing the nutrients with glucose‐d7 substitution. SRS images were acquired using a summed signal across the C–D vibrational region (2050–2300 cm^−1^). Scale bar: 10 µm. B) SIP‐SRS images showed *C. albicans* daughter cells had visibly weaker C–D signal in response to non‐deuterium media. The white arrows highlight representative daughter cells, which display reduced C–D signal intensity than mother cells. SRS images were acquired using a summed signal across the C–D vibrational region (2050–2300 cm^−1^). Scale bar: 10 µm. C) SIP‐SRS images of glucose‐d7 uptake in *C. albicans* during hyphae development. SRS imaging was performed by tuning the Raman shift to C–D vibration at ≈2150 cm^−1^. Scale bar: 10 µm. D) Fluorescence imaging illustration of the chitin and mannan layers in the hyphae form *C. albicans*. Scale bar: 10 µm. E) The cell wall synthesis dynamics during *C. albicans* hyphae growth under various nutrients environment supplemented with glucose‐d7 (D7) and/or glucose‐d0 (D0). The segmentation of the cell wall intensities in the hyphae tails is indicated by white arrows. SRS images were acquired by tuning the Raman shift to C–D vibration at ≈2150 cm^−1^. Scale bar: 10 µm.

To study the cell wall synthesis dynamics underlying the cell wall reprogramming in *C. albicans* cells, we first investigated the sources of metabolites using SIP‐SRS imaging in the C–D bond region (2050 to 2300 cm^−1^) for fungal cells cultured with stable isotope probe glucose‐d7 and nutrients. We examined glucose uptake and derived metabolism by feeding *C. albicans* SC5314 cells deuterated glucose‐d7, and by substitution with non‐deuterated glucose‐d0. SIP‐SRS images showed non‐detectable C–D signal in *C. albicans* cells treated with glucose‐d0, whereas cells treated with glucose‐d7 exhibited a much stronger C–D signal (**Figure** **2**A). After refreshing the nutrients with glucose‐d7 substitution, the daughter cells showed stronger cell wall signals than the mother cells. Interestingly, when we switch the nutrient sequence by feeding *C. albicans* cells deuterated glucose‐d7 first, followed by non‐deuterated glucose‐d0 media, the correlate carbohydrate content in *C. albicans* cells becomes reversed in the mother cells and daughter cells. (Figure 2B). Specifically, after switching to glucose‐d0 treatment, *C. albicans* daughter cells had visibly weaker C–D signal in response to non‐deuterium media, while the mother cells retained intensive C–D signal in glucose‐d7‐rich culture environment. Following the switch to glucose‐d0, newly synthesized daughter cells incorporate the non‐deuterated glucose and therefore exhibit weaker C–D signals compared to the pre‐existing mother cells, which had incorporated glucose‐d7. The white arrows in Figure 2B highlight representative daughter cells, which display lower C–D signal intensity than mother cells. This pattern is consistent with the expected metabolic labeling behavior and supports our interpretation of active cell wall synthesis in the daughter cells.

*C. albicans* is able to grow in yeast, pseudohyphal and hyphal forms, a trait linked to its ability to invade epithelial cells and cause tissue damage.^[^ ^20^ ^]^ We first evaluated whether glucose‐derived C–D bonds could be detected during the *C. albicans* hyphae growth (Figure 2C). The SIP‐SRS imaging revealed glucose‐d7 uptake during hyphae development by comparing the untreated control group. The single cell Raman spectra of the lipid droplets (LDs) and non‐LDs area in the hyphae cells are showing different chemical features in Figure S7 (Supporting Information). Further fluorescence imaging confirmed the distinct cell wall layers in hyphal‐form *C. albicans* (Figure 2D). The imaging clearly showed that chitin layer is located in the middle of the hyphae tail, while the mannan layer encapsulates the entire hyphae cell. Next, we examined the dynamics of cell wall synthesis during *C. albicans* hyphae growth under various nutrients environment supplemented with glucose‐d7 (D7) and/or glucose‐d0 (D0) (Figure 2E). The experimental protocol is detailed in supporting Figure S8 (Supporting Information). In the “D0–D0–D0–D0” treatment group, the glucose substitute in the nutrition media is glucose‐d0 for sequential four times. Notably, there are no obvious C–D signals in the *C. albicans* culture, representing the lack of deuterium in the environment. Interestingly, replacing the glucose substitute to D7 and switching again to D0, we found the segmentation of the cell wall intensities (indicated by white arrows) in the hyphae tails in the “D0–D7–D0–D7” treatment group, suggesting discontinuous deuterated cell wall synthesis. In these conditions, the average call wall C–D signals at 2160 cm^−1^ is roughly proportional to the percentage of deuterated glucose in the culture media. A similar segmentation cell wall pattern was observed in single hyphae‐form cells in the “D7–D0–D7–D0” treatment group (indicated by white arrows). However, the metabolic switch conditions reflect distinct temporal incorporation windows and corresponding differences in cell wall assembly dynamics. Notably, we observe clear differences in the spatial distribution of deuterium signals, particularly at the hyphal tail, suggesting differential patterns of cell wall remodeling over time between different nutrient conditions. Using SIP‐SRS imaging, we were able to evaluate the deuterium fraction with high specificity at the single‐cell level during in situ *C. albicans* hyphal growth. This labelling and imaging procedure is for general application to monitor live biomolecule synthesis.

### 2.3 Longitudinal SIP‐SRS Imaging of Cell Wall Synthesis Dynamics in Live Yeasts

**Figure 3:**
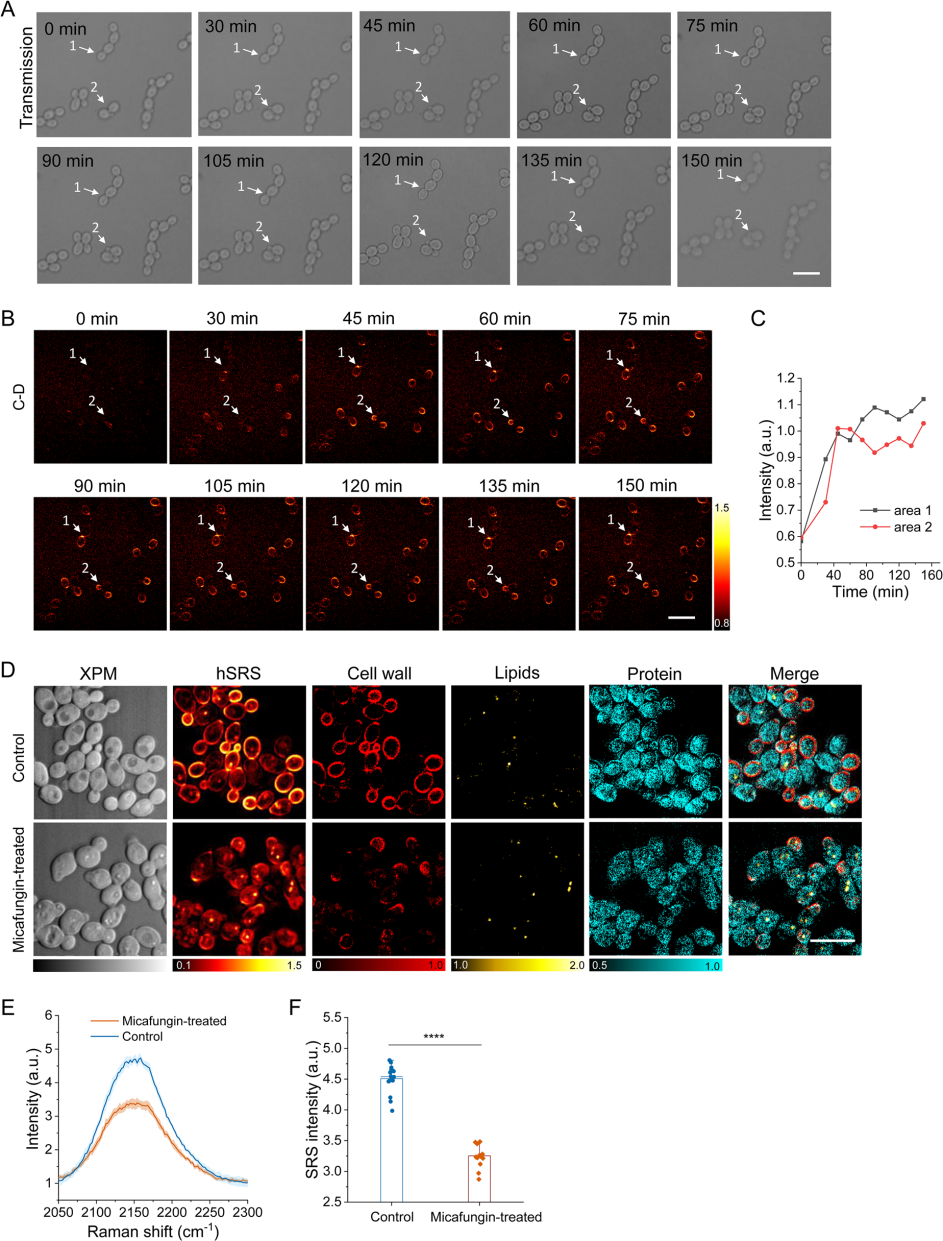
Longitudinal SIP‐SRS imaging of live yeast cell wall synthesis dynamics. A) sequential transmission images of single *C. albicans* SC5314 cells at specific time intervals ranging from 0 to 150 min. Scale bar: 10 µm. B) The longitudinal time‐resolved SIP‐SRS imaging of live yeast cell wall synthesis dynamics demonstrated increasing C–D signals, revealing the emergence and development of newly synthesized daughter cells over time. SRS images were acquired by tuning the Raman shift to C–D vibration at ≈2160 cm^−1^. Scale bar: 10 µm. C) Quantitative cell wall C–D intensities of individual *C. albicans* cells in A) and B), indicated by white arrows. D) The decomposed hyperspectral SRS images and the corresponding XPM images of *C. albicans* in response to micafungin treatment. LASSO unmixing was applied to visualize changes in cell wall, lipids and protein, allowing clearer differentiation of micafungin‐induced structural disruptions. Scale bar: 10 µm. E) Average SRS spectra of *C. albicans* after culture in normal and micafungin‐containing medium. F) Average SRS C–D intensity plot for *C. albicans* in (D) with N ≥ 10 per group. Significance was evaluated using an unpaired t‐test (***, *p* < 0.001).

To further validate the sensitivity and accuracy of SIP‐SRS imaging in monitoring the biomolecule synthesis dynamics, we obtained in situ biomass signals at the C–D vibrational bond region to reveal dynamic changes in the cell wall as it evolves over time. As shown in **Figure** **3**A, sequential images capture the in situ incorporation of specific deuterium markers into the cell wall matrix, highlighting changes in distribution and density in single *C. albicans* SC5314 cells at specific time intervals ranging from 0 to 150 min. By inspecting the time‐dependent transmission images and SIP‐SRS images, we targeted the newly formed daughter cells (indicated by white arrows). The time‐resolved SIP‐SRS imaging demonstrated increasing C–D signals during the time range, revealing the emergence and development of newly synthesized daughter cells over time (Figure 3B). In addition to spatial information, quantitative cell wall C–D intensities were obtained (Figure 3C). The C–D intensity increased drastically until ≈60 min, followed by a slower increase until 150 min. The C–D intensity increase, especially during the first 60 min, aligns with the kinetics of β‐1,3‐glucan and chitin deposition during bud growth, highlighting the spatial and temporal dynamics of wall biosynthesis. The growth rates of *C. albicans* show substantial variations according to the strain and culture conditions used. It is reported that under optimal conditions it can achieve maximal doubling times of just under 1 h.^[^ ^21^ ^]^

These time‐resolved SIP‐SRS images not only captured the in situ rearrangements of cell wall component during cell growth but also provide insight into the reaction dynamics of the biosynthetic pathway, specifically the timing, spatial localization, and intensity of cell wall macromolecule synthesis. Such visualizations offer invaluable insights into the kinetics of cell wall assembly, revealing how cells modulate the cell wall polysaccharide synthesis in response to environmental conditions. Taken together, real‐time SIP‐SRS imaging has revolutionized our ability to study cell wall synthesis by providing detailed, time‐dependent chemical imaging of this complex process. These results confirmed that the observed C–D signals originated from newly synthesized macromolecules. These data collectively showed that glucose‐d7 was actively harnessed for the growth of fungi and that the metabolic conversion rate could be quantified by SIP‐SRS imaging of individual fungal cells at the vibrational frequency of the C–D band.

### 2.4 SIP‐SRS Imaging Uncovers Cell Wall Remodeling Under Micafungin Treatment

By simultaneously mapping multiple metabolites through SIP‐SRS imaging, we can uncover alterations in the fungal cell metabolic network upon antifungal treatment. In response to micafungin treatment, a common inhibitor of β‐1,3‑glucan synthesis that disrupt fungal cell wall production, *C. albicans* SC5314 cells exhibited a considerable decrease in cell wall content and similar protein and LD levels compared with the untreated control group (Figure 3D). SIP‐SRS images show a pronounced reduction in the density, abnormal morphology, and uniformity of the cell wall matrix following micafungin treatment, indicating an inhibition of cell wall assembly and remodeling. LASSO unmixing further enhances the visualization of this effect, providing clearer differentiation of cell wall components and highlighting micafungin‐induced structural alterations. These imaging results demonstrate a clear impact of micafungin on cell wall synthesis, revealing a marked disruption in the incorporation of glucose components into the cell wall. The averaged SRS C–D spectra of *C. albicans* cells cultured in either normal medium or micafungin‐containing medium reveal that micafungin treatment leads to a reduction in C–D signal, suggesting decreased metabolic incorporation under drug stress (Figure 3E). The analysis of cell wall C–D signal intensity from individual cells shows a significant reduction in C–D intensity in the micafungin‐treated group compared to the untreated control, indicating suppressed metabolic activity (Figure 3F).

Based on the corresponding fluorescence imaging, the β‐1,3‑glucan marker did not show a distinct reduction in signal intensity in the micafungin‐treated *C. albicans* cells (Figure S9, Supporting Information). The difference between SRS and immunofluorescence signals is due to their detection principles. 1,3‐β‐glucan is a long polysaccharide composed of hundreds to thousands of glucose units. Inhibition of 1,3‐β‐glucan synthase by micafungin leads to shorter glucan chains, causing a strong reduction in SRS signal, as SRS measures total polymer content and scales with chain length. In contrast, immunofluorescence detects specific epitopes, which may remain on shorter glucan chains, resulting in only a modest change. Thus, SRS reflects polymer quantity, while immunofluorescence reflects epitope presence. Therefore, the reduction in fluorescence intensity following micafungin treatment was relatively subtle. SRS enables direct, chemical‐specific visualization of native cell wall components in *C. albicans* without the need for exogenous labels or probes, thus providing a more accurate and unbiased assessment of β‐glucan dynamics and cell wall remodeling in response to antifungal treatment. By providing real‐time, subcellular resolution visualizations, SIP‐SRS imaging elucidates the effects of micafungin on cell wall remodeling, revealing its targeted inhibition of cell wall biosynthesis. This visualization underscores micafungin's efficacy in targeting and impeding cell wall biosynthesis, offering a detailed understanding of its mechanism of action at the molecular level. Our findings are essential for understanding antifungal mechanisms and for informing the development of more effective therapeutic strategies.

### 2.5 SIP‐SRS Imaging Uncovers Cell Wall Remodeling Under AmB Treatment

**Figure 4:**
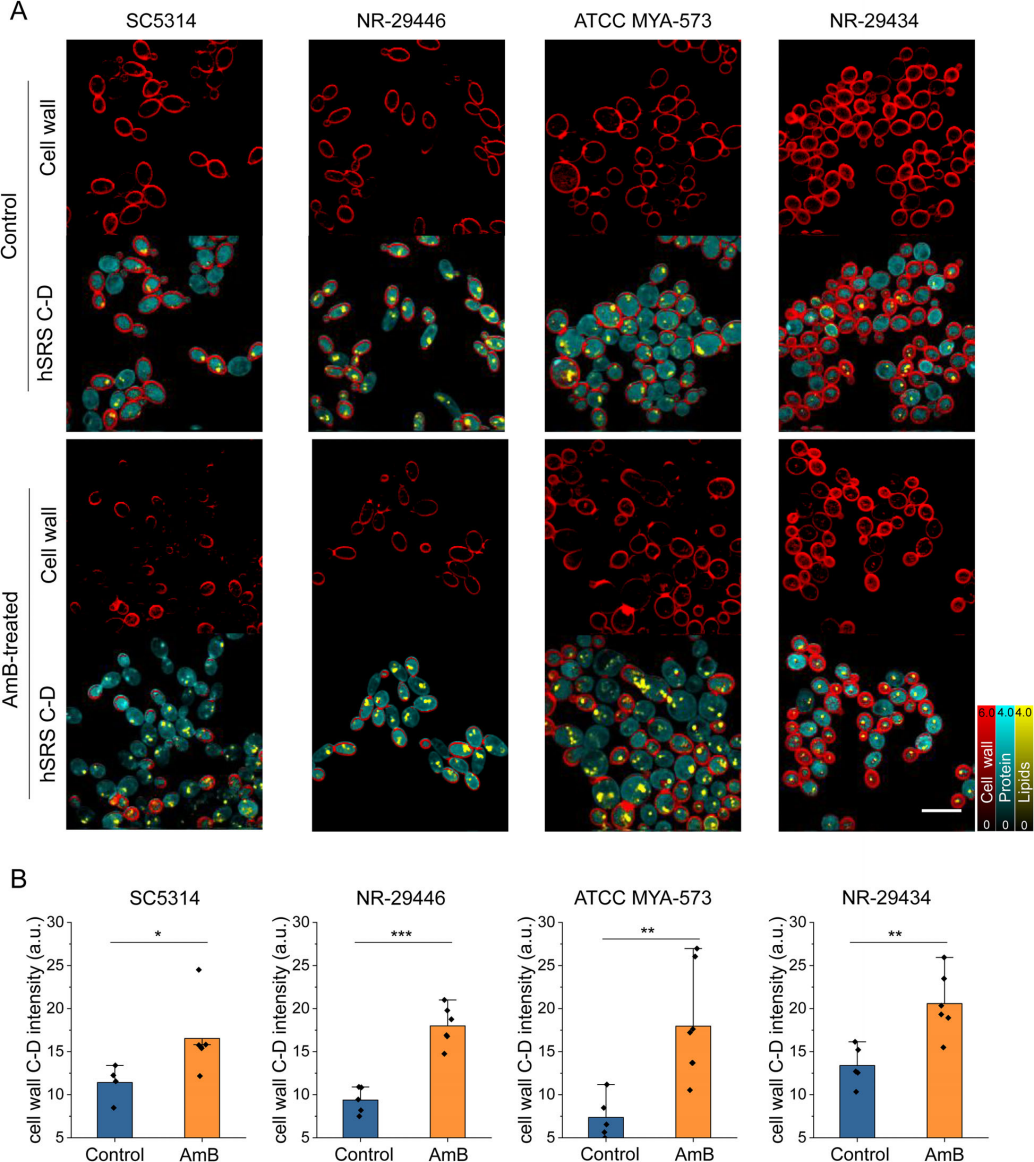
SIP‐SRS imaging of cell wall remodeling under AmB treatment. A) SIP‐SRS imaging of *C. albicans* in response to AmB treatment. Scale bar: 10 µm. B) The quantitative analysis showed significant increase in cell wall content intensity in *C. albicans*. Significance was evaluated using an unpaired *t*‐test (*, *p* < 0.05; **, *p* < 0.01; ***, *p* < 0.001). N ≥ 5 per group.

SIP‐SRS imaging has provided significant insights into the effects of AmB on cell wall remodeling, offering real‐time, high‐resolution observations of cellular processes. **Figure** **4**A shows SRS imaging results revealing that AmB treatment leads to a notable effect in cell wall synthesis dynamics in *C. albicans* SC5314, *C. albicans* NR‐29446, *C. albicans* ATCC MYA‐573, *C. albicans* NR‐29434. The AmB‐treated cells exhibited heterogeneity, and the average signal showed a modest decrease. Interestingly, after AmB treatment daughter cells showed increased cell wall intensity. This is evident from the enhanced density and uniform distribution of cell wall intensities in daughter cells following AmB exposure. The quantitative analysis showed significant increase in cell wall content intensity across all strains studied (Figure 4B). SIP‐SRS imaging was performed to assess metabolic responses of *C. auris* species to AmB treatment (Figure S10, Supporting Information). Overall, the C–D signal intensity was decreased in AmB‐treated cells, indicating suppressed biosynthetic activity. However, SIP‐SRS images revealed spatial differences within the cell population. Specifically, mother cells showed a marked reduction in C–D intensity, particularly in the cell wall and cytoplasmic regions, suggesting strong metabolic inhibition. In contrast, daughter cells retained relatively higher cell wall C–D signals, indicating more active cell wall metabolism compared to the mother cells. This differential response may reflect a stress‐adaptive mechanism, where daughter cells maintain higher biosynthetic activity to support survival or recovery under antifungal pressure. The segmented SRS maps further highlight these differences by resolving distinct cell wall, lipid, and protein distributions across individual cells. These observations suggest that AmB stimulates an upregulation of cell wall biosynthesis, potentially as a compensatory response to drug‐induced stress. Such findings provide valuable insights into the dual effects of AmB, highlighting its role in modulating cell wall dynamics and guiding future research into its therapeutic applications and mechanisms of action.

### 2.6 The Synergy between AmB and Micafungin

**Figure 5:**
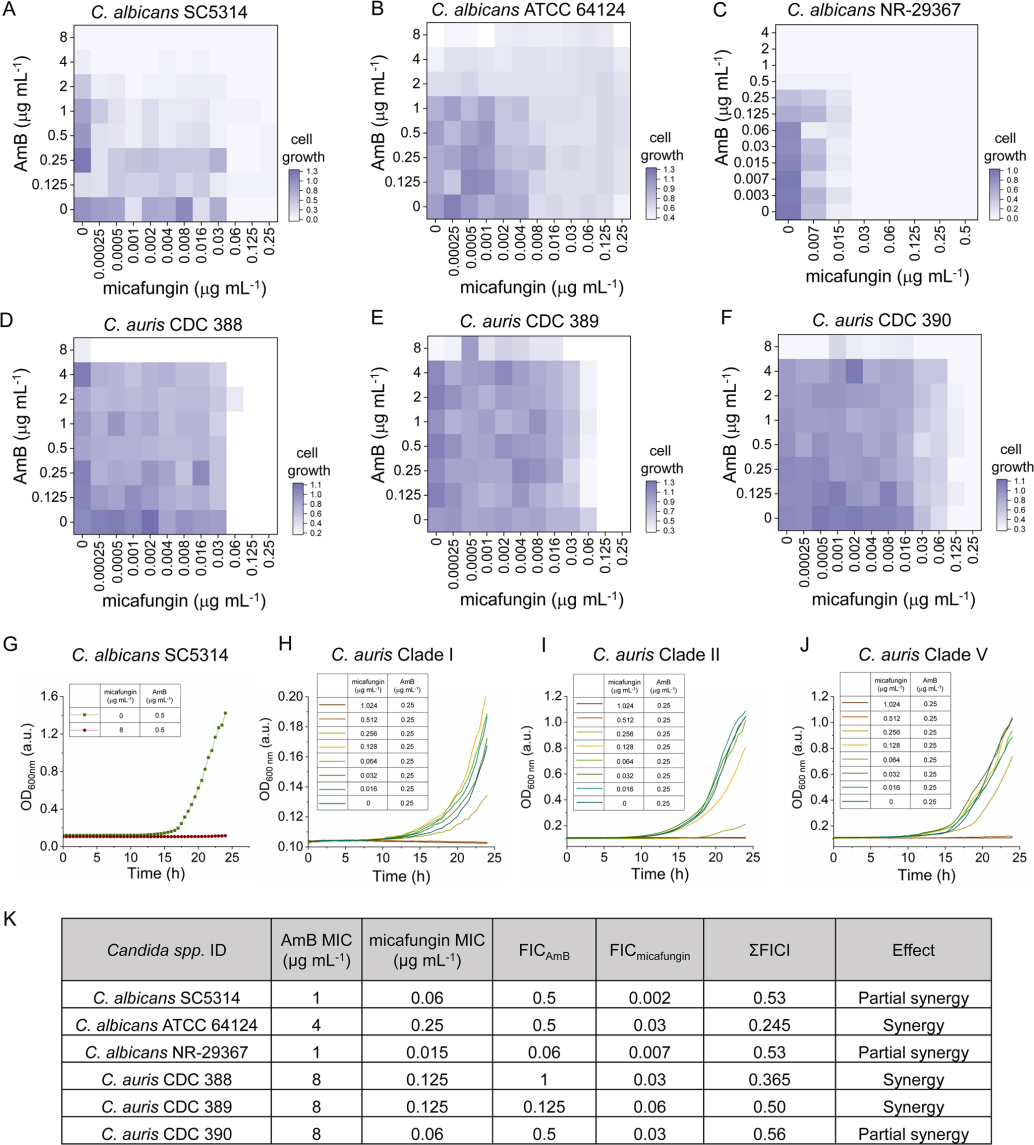
The synergistic effect of micafungin and AmB on inhibition of fungal growth. A–F) A synergistic relationship between micafungin and AmB was determined in various *C. albicans* and *C. auris* strains. G–J) The growth curves of the *C. albicans* SC5314, *C. auris* Clade I, *C. auris* Clade I and *C. auris* Clade V illustrate the synergistic impact of AmB and micafungin on the cell viability. (K) Fractional inhibitory concentration index (ΣFICI) of micafungin with AmB treatment in *C. albicans* strains and *C. auris* strains. ΣFICI ≤ 0.5: synergy; 0.5–0.75: partial synergy.

After confirming the efficacy of micafungin treatment in *Candida* species cells, and the SIP‐SRS imaging predictions that AmB treatment alters the cell wall synthesis, we investigated the synergistic effect of combining micafungin and AmB on fungal inhibition. This synergy was compellingly demonstrated through both SIP‐SRS imaging and biological checkerboard assays. Checkerboard assay by measuring the optical density of fungal growth vividly depicts how the combination of AmB and micafungin results in a more pronounced inhibition of cell viability compared to individual treatments. We performed a viability test on various *C. albicans* cells treated with AmB and micafungin, including *C. albicans* SC5314, *C. albicans* ATCC 64124, *C. albicans* NR‐29367 (**Figure** **5**A–C), and *C. auris* species *C. auris* CDC 388, *C. auris* CDC 389, and *C. auris* CDC 390 (Figure 5D–F). The growth curves of the *C. albicans* SC5314 (Figure 5G), *C. auris* Clade I (Figure 5H), *C. auris* Clade II (Figure 5I) and *C. auris* Clade V (Figure 5J) illustrate the synergistic impact on the cell viability, with a various concentration of AmB and micafungin treatment. Additionally, the combination of micafungin (at 8 µg mL^−1^) with AmB (at 0.5 µg mL^−1^), reduced the growth of *C. albicans* wild type as observed over a 24‐hr period. The calculation of fractional inhibitory concentration index (ΣFICI) confirmed the synergistic effect between AmB and (Figure 5K). Interestingly, the combination of micafungin and AmB reduced the minimum inhibitory concentration (MIC) of AmB in all tested isolates to sensitive levels. Complementary to the SIP‐SRS imaging results, biological checkerboard assays confirm the synergistic interaction between AmB and micafungin, quantitatively validating the enhanced antifungal activity observed. The combination treatment of micafungin and AmB on *Candida species* had a crucial synergistic effect in supressing cell growth and reducing MICs. These assays reveal a marked decrease in the MICs of each drug when used in combination, further supporting the observed synergy and highlighting the potential of this combinatorial approach to improve therapeutic outcomes against fungal infections.

## 3 Discussion

Invasive fungal infections are serious and often life‐threatening diseases that have been underestimated for a long time. Despite their significant impact, fungal infections remain among the least discussed public health threats, even though they claim around 1.5 million lives each year.^[^ ^6a^ ^]^ The global burden of fungal infections has risen noticeably in recent decades, driven by the increasing number of vulnerable individuals, including patients with AIDS, cancer, or those undergoing chemotherapy or organ transplantation.^[^ ^6^, ^22^ ^]^

AmB, introduced in the 1950s, remains the most comprehensive systemic antifungal agent and is considered the gold standard for treating life‐threatening invasive fungal infections. While AmB is highly effective as an antifungal and antiprotozoal agent, its clinical application is limited by significant drawbacks and serious dose‐limiting side effects.^[^ ^23^ ^]^ Compounding this issue, 30% of *C. auris* isolates now exhibit resistance to AmB.^[^ ^24^ ^]^ Combining AmB with potent enhancers could be an effective alternative to overcome resistance without rising AmB toxicity.

In this study, we harness SIP‐assisted hyperspectral SRS microscopy (SIP‐SRS) to directly visualize and interrogate the dynamics of fungal cell wall synthesis under antifungal treatment. By leveraging the enhanced sensitivity and spatial resolution of SIP‐SRS, we undercover previously inaccessible details of fungal metabolism and drug interactions. Our findings demonstrate a synergistic inhibition of fungal growth by AmB and micafungin, antifungals from distinct classes, highlighting the therapeutic promise of combined antifungal strategies. This approach not only advances our understanding of fungal physiology at the molecular level but also opens promising avenues for combating drug‐resistant fungal infections. Our study sheds light on the complex interaction between antifungal medications and fungal cellular responses, aiming to develop more targeted and effective treatments against multi‐drug resistant fungi.

The imaging results presented offer significant insights into the dynamics of cell wall synthesis and the impact of antifungal treatments on this crucial biological process. SIP‐SRS imaging has provided a powerful visualization tool, revealing detailed, real‐time information about cell wall remodeling under various conditions. The use of D_2_O in previous studies was instrumental in tracing cell wall biosynthesis, but the advantages of using glucose‐d7, as demonstrated in our current findings, offer superior resolution and specificity. Glucose‐d7, a stable isotope‐labeled glucose, enhances the detection of newly synthesized cell wall components, providing a more accurate and dynamic view of cell wall formation and turnover. This refinement is particularly useful in understanding the subtle changes in cell wall dynamics induced by antifungal treatments. Furthermore, discrimination between chitin, mannan, and β‐glucan, as well as resolving intracellular protein structures, could be further improved by combining high‐numeric‐aperture objective imaging with advanced machine learning classification, which warrants further investigation in future work.

Our findings also underscore the importance of investigating biofilm formation and growth. Biofilms represent a significant challenge in fungal infections due to their increased resistance to antifungal therapies. The SIP‐SRS imaging is capable of visualizing potential implications for treatment strategies. Notably, the synergistic effect of AmB and micafungin observed in our studies may offer new avenues for disrupting biofilm formation, thereby enhancing therapeutic efficacy. Due to the pronounced adverse reactions associated with AmB, the recommended plasma concentration in clinical settings is limited to 1–2 µg mL^−1^. As a result, its effectiveness is significantly compromised when treating *Candida* strains with MIC values exceeding 1 µg mL^−1^.^[^ ^25^ ^]^ With the global prevalence of *C. auris* infections on the rise, significant efforts are focused on preserving AmB as a last‐resort antifungal agent. In this study, we aimed to overcome *C. auris* resistance to AmB by employing a combinatorial strategy with micafungin and directly visualizing their interaction. Our in vitro results revealed that this combination was effective against 100% of all tested isolates: *C. albicans* SC5314, *C. albicans* ATCC 64124, *C. albicans* NR‐29367, and *C. auris* species *C. auris* CDC 388, *C. auris* CDC 389, and *C. auris* CDC 390.

The potential of these findings can be extended, particularly in rapid antifungal susceptibility testing (AFST). The ability to visualize real‐time changes in cell wall synthesis and biofilm dynamics can significantly accelerate the identification of effective antifungal treatments. By integrating advanced imaging techniques with rapid AFST methods, clinicians could make more informed decisions on antifungal therapy, leading to improved patient outcomes and more effective management of fungal infections. Overall, these advancements highlight the critical role of advanced imaging in elucidating drug mechanisms and optimizing therapeutic strategies.

## 4 Experimental Section

### Spectroscopic Stimulated Raman Scattering Imaging System

The spectral focusing approach was used to recover spectral resolution, where the Raman shift was tuned by controlling the temporal delay between 2 chirped femtosecond pulses.^[^ ^26^ ^]^ A femtosecond laser operating at 80 MHz was used to generate the pump and Stokes laser source (InSight DeepSee, Spectra‐Physics). One output beam was turntable from 680–1300 nm wavelength, which served as the pump beam. The other beam at 1040 nm served as the Stokes beam. The acoustic‐optical modulator (AOM, 1205‐C, Isomet) was used to modulate the Stokes beam at 2.4 MHz. A dichroic mirror w used to collinearly combine the pump and Stokes laser beams. The combined beam was chirped by SF57 glass rods, and was sent into a laser‐scanning microscope via a 2‐D galvo mirror. The laser was focused on the specimen via a 60× water objective (NA = 1.2, UPlanApo/IR, Olympus) and collected via an oil condenser (NA = 1.4, U‐AAC, Olympus). In order to collect the SRS signals from the pump beam, a filter (1000SP, Thorlabs) was used to filter out Stokes signals. A photodiode with a custom‐built resonant circuit was used to collect signals. The final SRS signal was extracted by a lock‐in amplifier (HF2LI, Zurich Instrument) and was digitized via a high‐speed data acquisition card (PCIe 6363, National Instrument). The temporal delay timing was controlled by an automatic stage that moved forward with a step size of 10 µm.

To obtain the hyperspectral stimulated Raman scattering (hSRS) metabolic images of fungi at the C–D vibrational frequency, the pump wavelength was tuned to 849 nm with an output power of ≈30 mW. For C–H vibrational frequency, the pump wavelength was tuned to 798 nm. Meanwhile, the output Stokes power was set at ≈300 mW for C–D vibrational imaging, or ≈100 mW for C–H vibrational imaging. Each image contained 200 × 200 pixels.

### Chemicals and Reagents

Yeast extract peptone dextrose (YPD), D_2_O, formalin solution (10%, neutral buffered), calcofluor white (CFW) were purchased from Sigma‐Aldrich. Concanavalin A (Invitrogen), Phosphate‐buffered saline (PBS), Roswell Park Memorial Institute 1640 (RPMI 1640) Medium, 3‐(N‐morpholino) propane sulfonic acid (MOPS), and Y‐PER reagent were purchased from Thermo Fisher Scientific.

### Biological Specimens and Cell Culture Conditions

Fungal isolates were initially revived and cultured in a sterile YPD agar plate at 30 °C. Then, fungal isolates were cultured in sterile YPD broth at 30 °C in an orbital shaker (VWR, model 3500I) at a shaking speed of 200 rpm at a tilted angle of 45°. Logarithmic‐phase cells were harvested, centrifuged, and then diluted to a concentration at 10^6^ CFU ml^−1^ into glucose‐d7‐containing RPMI 1640 medium for metabolic incorporation. To prepare the RPMI 1640 medium, MOPS was used to adjust the pH value of the medium solution to 7.0. The final solution was sterilized by filtering using a 200 nm filter.

After incubation in glucose‐d7 (2%, w/v) and/or antifungal containing RPMI 1640 medium for 1 h, cells were centrifuged, washed with fresh 1×PBS, and then fixed in 10% formalin solution. The fungal specimen was washed twice using 1×PBS before imaging. Cells were sandwiched between 2 cover glasses (VWR International).

In the *C. albicans* hyphal incubation experiment, wild‐type cells were first cultured overnight in RPMI medium containing either glucose‐d7 (2%, w/v) or glucose‐d0 (2%, w/v). After adjusting the cell concentration to 1 × 10^6^ CFU mL^−1^, the cells were seeded into petri dishes and incubated for a defined period, followed by a nutrient switch (Figure S8, Supporting Information). The protocol consisted of an initial 1 h 20 min incubation, followed by two sequential 20‐min treatments with either glucose‐d7 or glucose‐d0. Cells were then harvested, washed with PBS, and fixed for subsequent imaging.

In the antifungal treatment study, *C. albicans* or *C. auris* cells were treated with micafungin (8 µg mL^−1^) or AmB at their respective MIC concentrations for 2 h, followed by supplementation with glucose‐d7 for an additional 45 min. Cells were then collected, washed, and prepared for subsequent imaging analysis.

### Antifungal Susceptibility Testing

Antifungal susceptibility testing was performed using the classical microdilution method, employing two‐fold serial dilutions of various antifungals according to current CLSI guidelines M27 and M38‐A2.^[^ ^27^ ^]^ Two‐fold serial dilutions of antifungal agents and fungal suspensions in sterile RPMI 1640 medium were added to a 96‐well plate. The optical densities were measured by a microdilution plate reader (Molecular Devices) in flat‐bottom microplates at 600 nm after 24 h incubation at 30 °C. The minimum inhibitory concentration (MIC) values for micafungin was determined as the lowest drug concentration that produced at least a 50% decrease in cell growth compared to that of the drug‐free well. The MIC value for amphotericin B was the concentration that produced a 100% decrease in fungal growth.

### Checkerboard Broth Dilution Assays

To evaluate the combinatorial behavior between antifungals, checkerboard broth dilution assays was performed to calculate the fractional inhibition centration index. Logarithmic‐phase *C. albicans* cells were transferred to a 96‐well plate containing two‐fold dilution of AmB starting at 4 or 8 µg mL^−1^, followed by two‐fold dilution of micafungin starting at 0.25 or 0.5 µg mL^−1^ in a perpendicular direction. Then the plate was cultured at 30 °C for 24 h. The optical density at 600 nm (OD 600 nm) was recorded to represent the fungal cell number. A heat map correlated with OD 600 nm was generated to calculate the FICI.

### Spontaneous Raman spectroscopy

Raman spectra of individual fungal cells were obtained using a confocal Raman spectrometer with a 532 nm excitation laser (LabRAM HR evolution, Horiba scientific). The acquisition time for each spectrum from individual fungal cells was 30 s, with the laser power at the sample maintained at approximately 10 mW.

### Macromolecule Isolation from Glucose‐d7 Treated Cells

To isolate macromolecules from fungal cells, Y‐PER protein extraction reagent was utilized separate and extract fungal cell proteins. *C. albicans* SC5134 cells were incubated in glucose‐d7 containing YPD medium for overnight and then were harvested and fixed with 10% neutral‐buffered formalin for 15 min, followed by three washes with PBS. Cells were then centrifuged, and pellets were resuspended in Y‐PER reagent and homogenized to release macromolecules. Following by incubation at room temperature for ≈20 min, debris were then spin down. The resulting supernatant is a concentrated protein solution.

### Image Processing and Single Cell Analysis

In the imaging analysis, the thresholding value in all SRS images was determined by the signal intensity in the negative control group. A C–D signal was considered negligible if its intensity was comparable to or lower than the cross‐phase modulation (XPM) background in the same spectral region. This criterion represents a thresholding approach, with the signal‐to‐background ratio showing a mean value of 1.0668 and a standard deviation of 0.0310 (Figure S2, Supporting Information). A pixel‐wise least absolute shrinkage and selection operator (LASSO) regression algorithm was employed to unmix the hyperspectral SRS images. The reference spectral profiles were obtained from hyperspectral SRS images of glucose‐d7‐treated cells, where the D‐protein spectrum was extracted from isolated cellular protein, while the D‐lipid and D‐cell wall spectra were derived by averaging signals from corresponding regions in the hyperspectral SRS imaging data which were normalized using the corresponding XPM background spectrum. To analyze the SRS signal intensity of cell wall, the SRS signal across the cell periphery was integrated and quantified using the LASSO‐unmixed cell wall maps. The Student's unpaired t‐test was used to determine whether there was any statistically significant difference between treatment groups (*, *p* < 0.05; **, *p* < 0.01; ***, *p* < 0.001; ****, *p* < 0.0001; n.s., not significant.). Data visualization was done using ImageJ.

### Fluorescence Imaging

Fungal cells were fixed in 4% paraformaldehyde, washed with PBS, and optionally blocked with 1% BSA. To stain mannan layer of the cell wall, fluorescein‐conjugated concanavalin A (ConA‐FITC) was used to bind specifically to α‐mannopyranosyl and α‐glucopyranosyl residues. Cells were incubated with ConA‐FITC (100 µg mL^−1^) for 30 min at room temperature in the dark. To stain mannan layer of the cell wall, CFW was employed to bind chitin and cellulose. Cells were incubated with CFW (50 µg ml^−1^) for 10 min and washed with PBS. To detect β‐1,3‑glucan, a β‐1,3‑glucan‐specific monoclonal antibody (mouse anti‐β‐1,3‐glucan) was applied, followed by incubation with a fluorescently labeled secondary antibody (Alexa Fluor 488 anti‐mouse IgG). Primary antibody incubation was done for 1 h at room temperature or overnight at 4 °C, followed by PBS washes and secondary antibody incubation for 30–60 min in the dark. Following removal of the secondary antibody solution, cells were washed three times with PBS in the dark and prepared for fluorescence imaging.

## Conflict of Interest

The authors declare no conflict of interest.

## Author Contributions

M.Z. and Y.Z. contributed equally to this work. M.Z. and J.‐X.C conceived the idea; M.N.S. provided the clinical fungal isolates and constructive discussions; M.M. provided constructive discussions; M.Z. and Y.Z. designed, performed, and analyzed initial SRS, fluorescence imaging experiments, in vitro mechanism studies, and synergistic therapy studies; H.L. developed the hyperspectral SRS unmixing method; J.C. helped with the cell culture studies; J.‐X.C. supervised the overall project; M.Z. and J.‐X.C. co‐wrote the manuscript; M.N.S. and M.M. revised the manuscript. All authors read and commented on the manuscript.

## Supporting information



Supporting Information

## Data Availability

The data that support the findings of this study are available from the corresponding author upon reasonable request.
