# Supplementary material for: SIP‐SRS Imaging of Cell Wall Synthesis Identifies a Synergy between Micafungin and Amphotericin B
Source: Adv Sci (Weinh). 2025 Oct 21;12(48):e07331. doi: 10.1002/advs.202507331 (PMC12752626; doi:10.1002/advs.202507331)
Supplement: Supplementary file 1 — Supporting Information [file ADVS-12-e07331-s001.docx]

Copyright WILEY-VCH Verlag GmbH & Co. KGaA, 69469 Weinheim, Germany, 2022.

Supporting Information

SIP-SRS Imaging of Cell Wall Synthesis Identifies a Synergy between Micafungin and Amphotericin B

Meng Zhang^#^, Yuewei Zhan^#^, Haonan Lin, Jiyang Chen, Mohamed N. Seleem*, Michael Mansour*, Ji-Xin Cheng*

*Corresponding authors: Mohamed N. Seleem ([seleem@vt.edu](mailto:seleem@vt.edu)), Michael Mansour (mkmansour@mgb.org), Ji-Xin Cheng ([jxcheng@bu.edu](mailto:jxcheng@bu.edu)).

# These authors contributed equally.

**This file includes:**

Figures S1 to S10

Supporting Figures


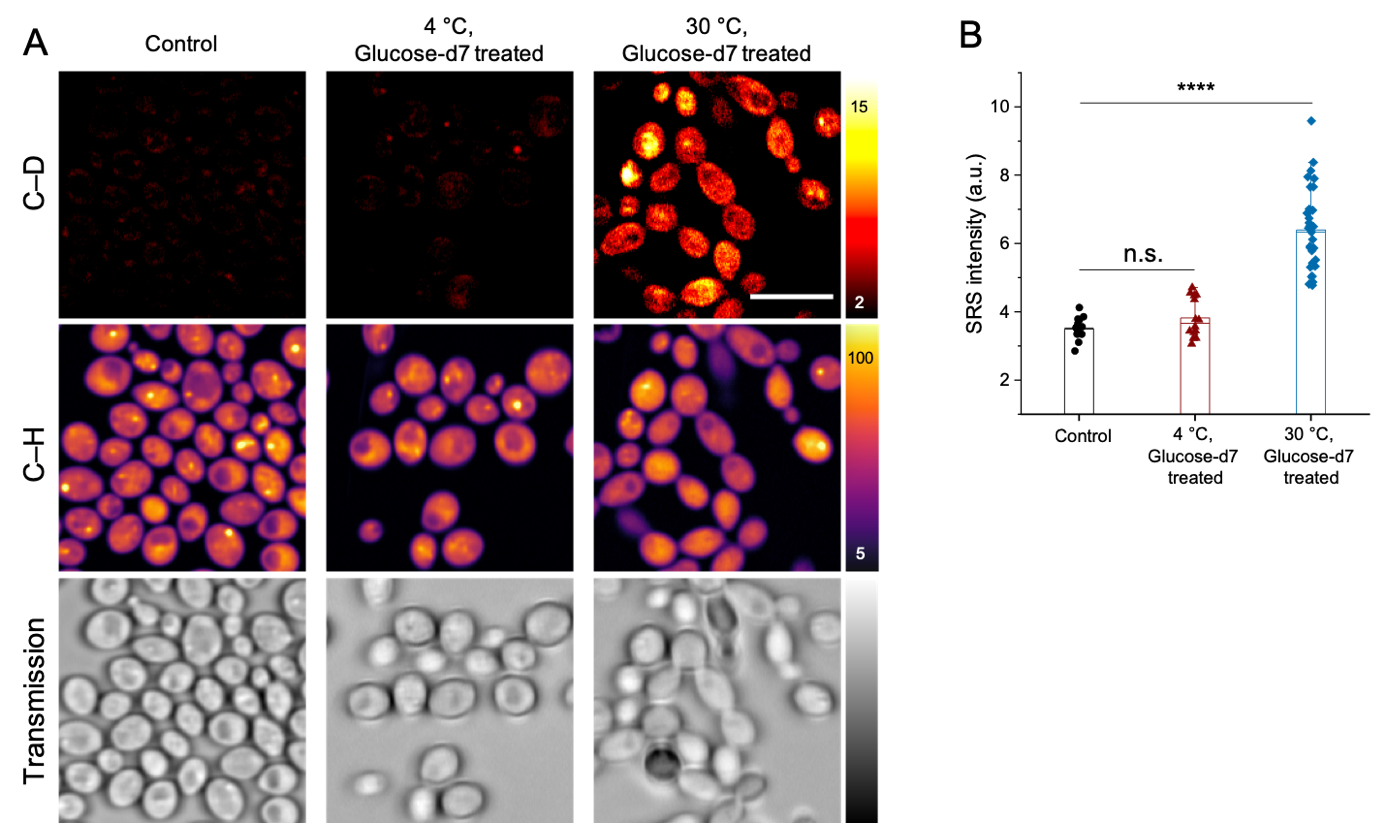


Supplementary Figure S1. (A) SRS imaging of *C. albicans* SC5314 showing both C–D and C–H channels, along with corresponding transmission images. Cells were cultured for 4 hours at either 4 °C or 30 °C in glucose-d7-substituted medium, with a control group cultured without glucose-d7. Scale bar: 10 µm. (B) Quantitative analysis of SRS C–D intensities from panel (A). N ≥ 20 per group. Significance was evaluated using an unpaired t-test (****, p < 0.0001; n.s., not significant).


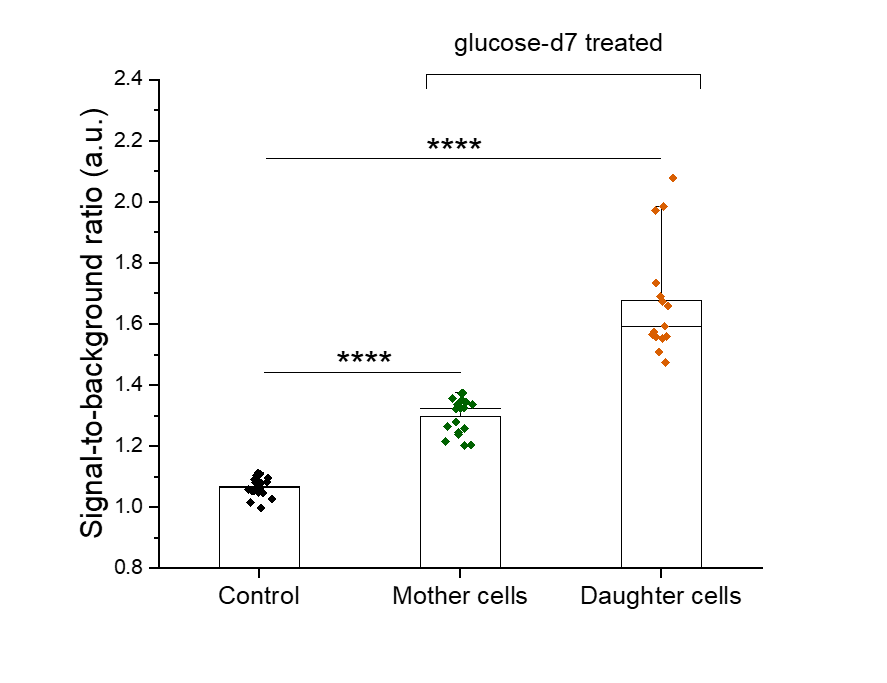


Supplementary Figure S2. Signal-to-background ratio for SRS microscopic C–D signal of individual *C. albicans* cells in Figure 1C. N ≥ 15 per group. Significance was evaluated using an unpaired t-test (****, p < 0.0001).


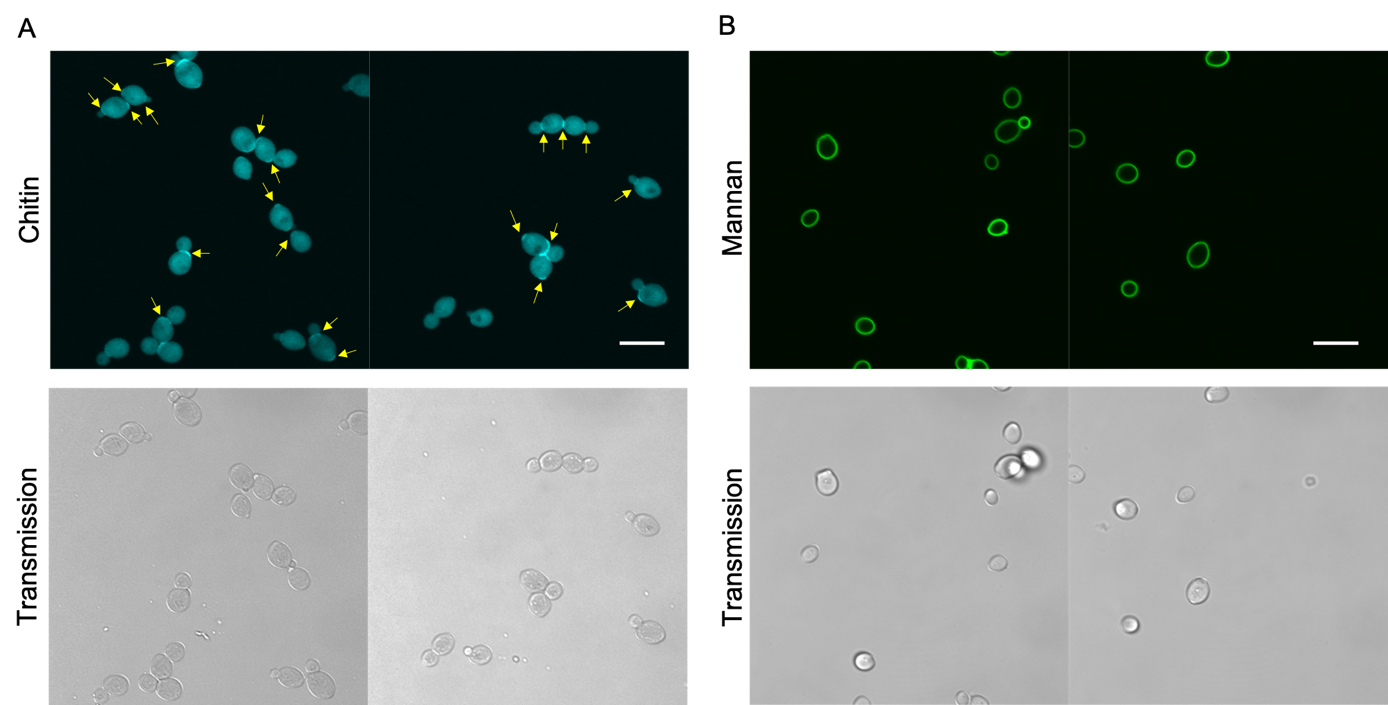


Supplementary Figure S3. Fluorescence imaging of (A) chitin, (B) mannan in *C. albicans*. Scale bar: 10 µm.


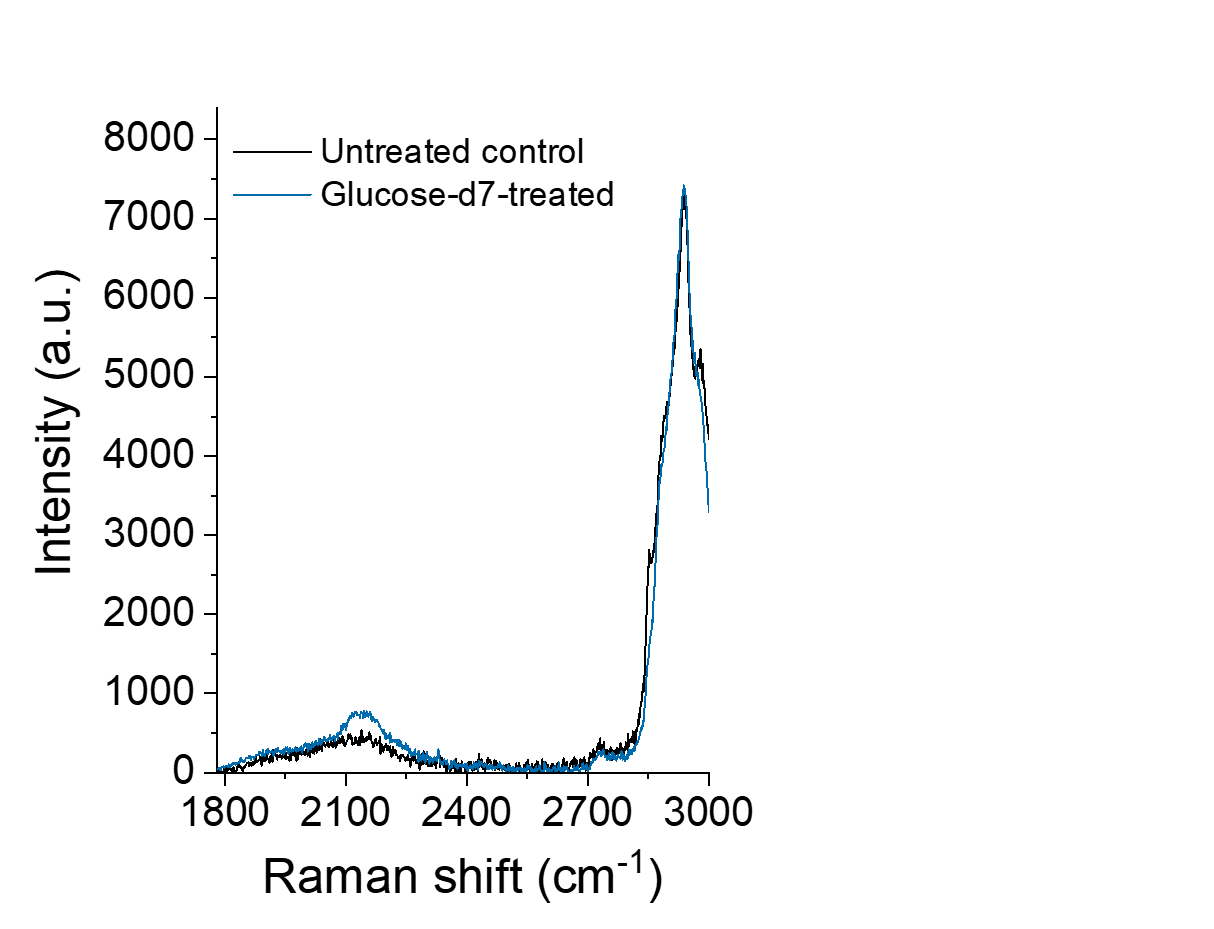


Supplementary Figure S4. Spontaneous Raman spectra show the C–D biosynthesis in *Candida albicans*. A broad peak (2050–2300 cm^-1^) at C–D vibration is distinct in glucose-d7 treated *C. albicans* SC5314.

**
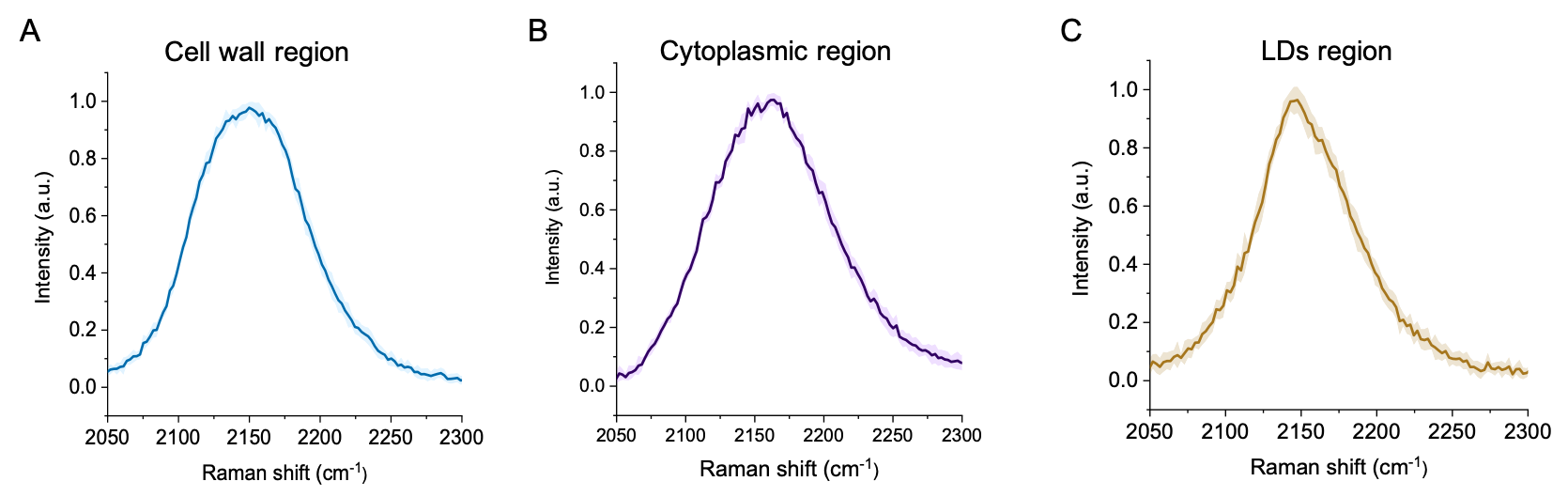
**

Supplementary Figure S5. Average single cell Raman spectra from (A) the cell wall region, (B) cytoplasmic region, and (H) the lipid droplets regions in *C. albicans* via glucose-d7 metabolism. N ≥ 15 per group.


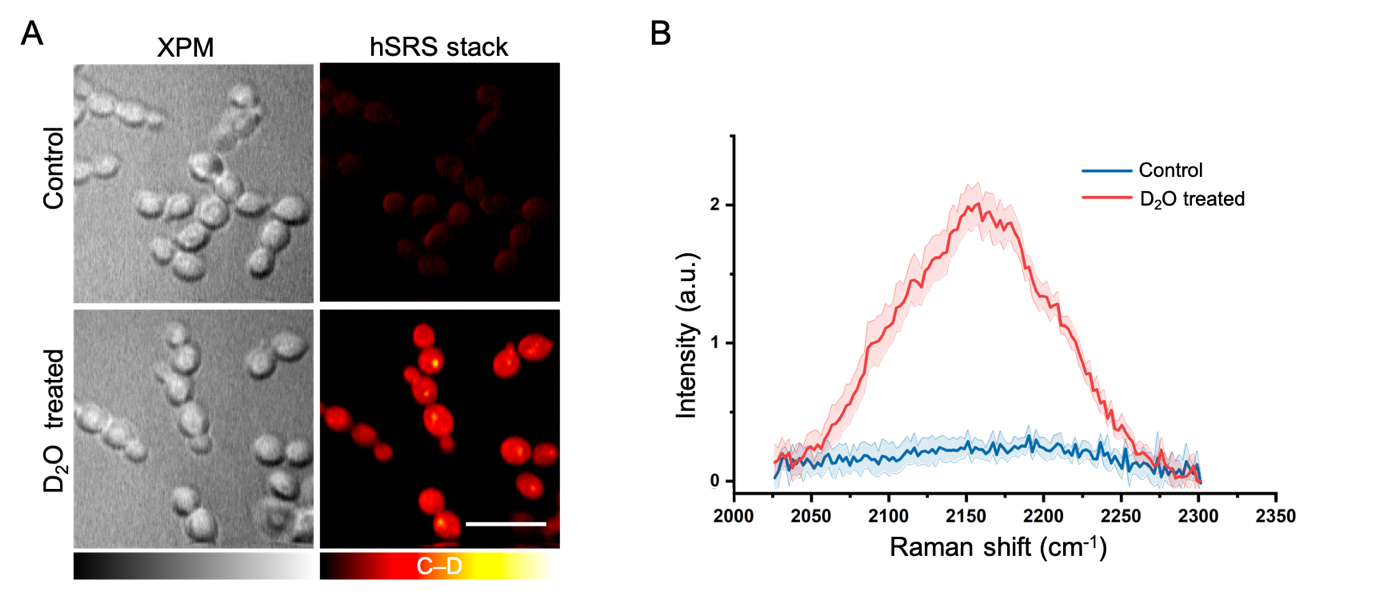


Supplementary Figure S6. Subcellular components by hyperspectral SRS imaging of C–D region. (A) SRS and corresponding XPM images of *C. albicans* after culture in normal and D_2_O-containing LB medium for 2 h. (B) SRS spectra of *C. albicans* after culture in normal and D_2_O containing LB medium for 2 h. N ≥ 15 per group. Scale bar: 10 µm.


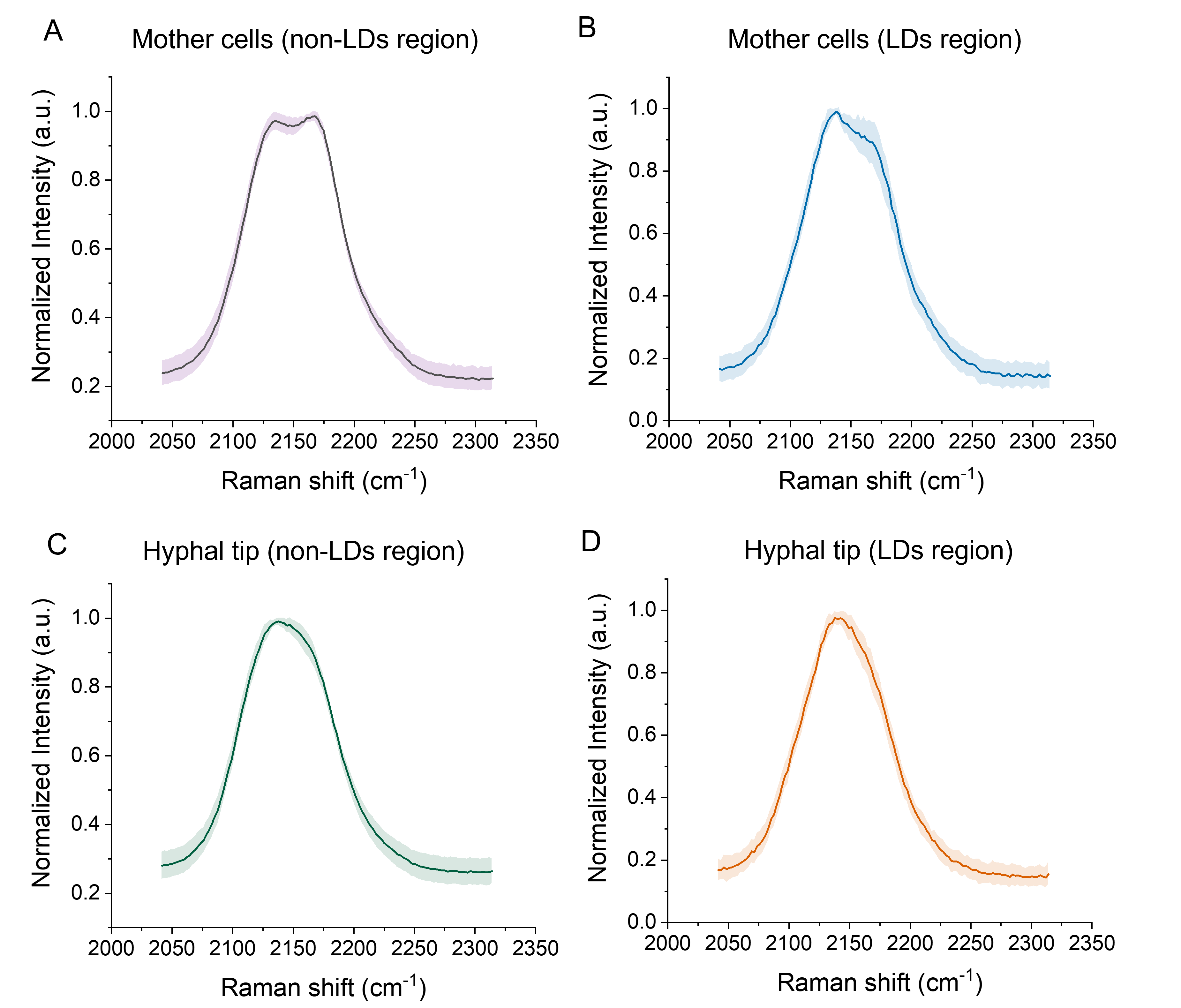


Supplementary Figure S7. Subcellular components by hyperspectral SRS imaging of *C. albicans* hyphal growth at C–D region. Hyperspectral SRS spectra of (A) mother cells in non-LDs regions; (B) mother cells in LDs regions; (C) the hyphal tip in non-LDs regions; (D) the hyphal tip in LDs region. N ≥ 15 per group.


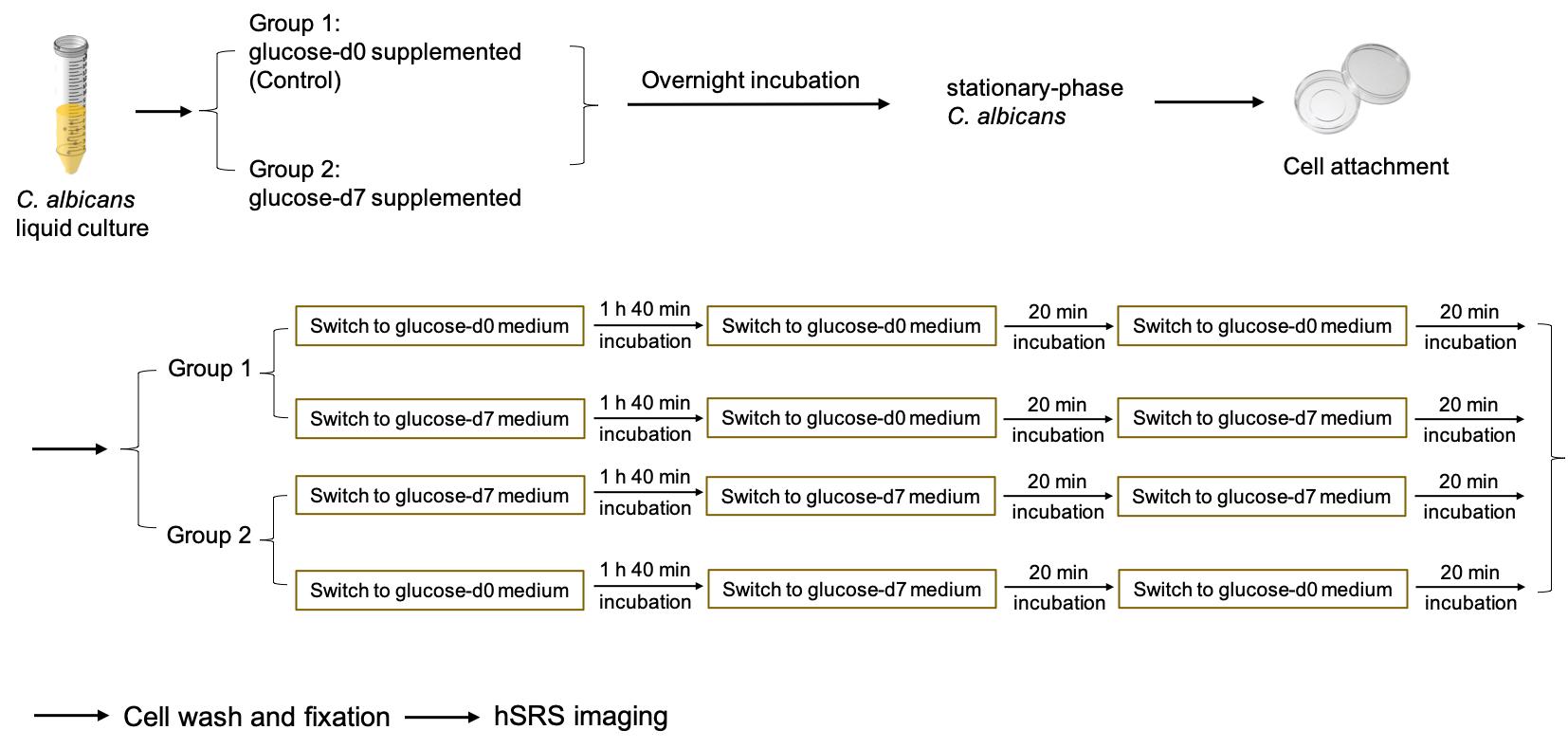


Supplementary Figure S8. Schematic illustration of *C. albicans* hyphae incubation for monitoring cell wall synthesis dynamics by glucose-d7 incorporation.


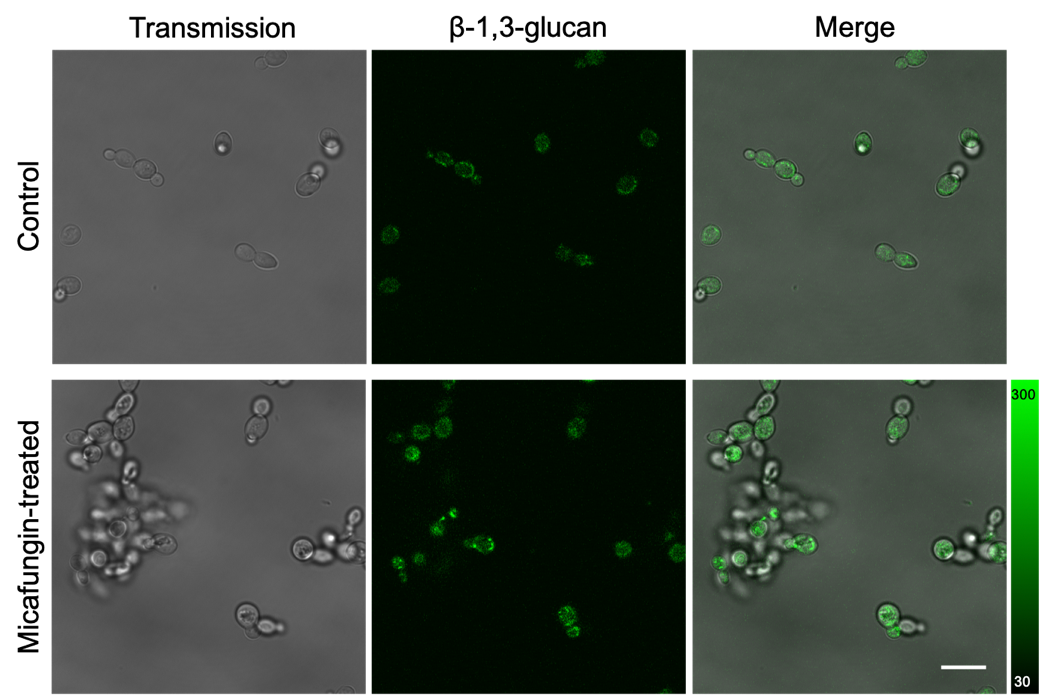


Supplementary Figure S9. Fluorescence imaging of the β-1,3‑glucan in *C. albicans* cells in response to micafungin treatment. Scale bar: 10 µm.


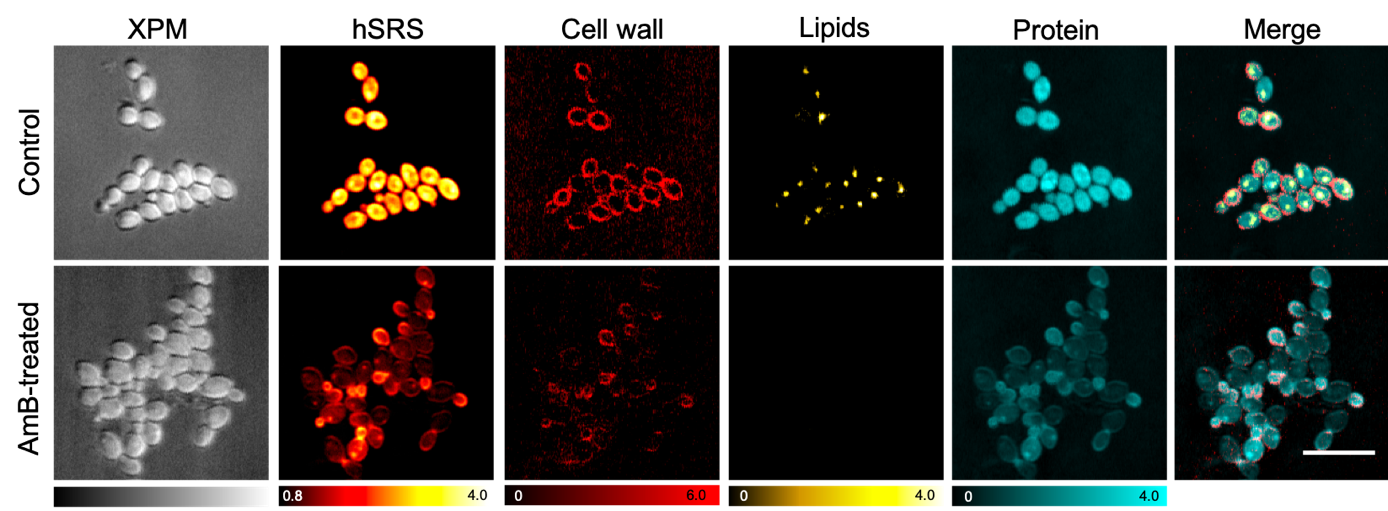


Supplementary Figure S10. SIP-SRS imaging of *C. auris* Clade V in response to AmB treatment. Representative hSRS image, mapped cell wall, lipids, and protein images. Scale bar: 10 µm.
